# Linking the International Wheat Genome Sequencing Consortium bread wheat reference genome sequence to wheat genetic and phenomic data

**DOI:** 10.1186/s13059-018-1491-4

**Published:** 2018-08-17

**Authors:** Michael Alaux, Jane Rogers, Thomas Letellier, Raphaël Flores, Françoise Alfama, Cyril Pommier, Nacer Mohellibi, Sophie Durand, Erik Kimmel, Célia Michotey, Claire Guerche, Mikaël Loaec, Mathilde Lainé, Delphine Steinbach, Frédéric Choulet, Hélène Rimbert, Philippe Leroy, Nicolas Guilhot, Jérôme Salse, Catherine Feuillet, International Wheat Genome Sequencing Consortium, Etienne Paux, Kellye Eversole, Anne-Françoise Adam-Blondon, Hadi Quesneville

**Affiliations:** 1 URGI, INRA, Université Paris-Saclay, 78026 Versailles, France; 2 International Wheat Genome Sequencing Consortium (IWGSC), 18 High Street, Little Eversden, Cambridge, CB23 1HE UK; 3 GDEC, INRA, Université Clermont Auvergne, 63000 Clermont-Ferrand, France; 4 Present address: GQE-Le Moulon UMR 320, INRA, Université Paris-Sud, Université Paris-Saclay, CNRS, AgroParisTech, Ferme du Moulon, 91190 Gif-sur-Yvette, France; 5 Present address: Inari Agriculture, 200 Sydney Street, Cambridge, MA 02139 USA; 6 International Wheat Genome Sequencing Consortium (IWGSC), 2841 NE Marywood Ct, Lee’s Summit, MO 64086 USA; 7 International Wheat Genome Sequencing Consortium (IWGSC), 5207 Wyoming Road, Bethesda, Maryland 20816 USA

**Keywords:** Data integration, Information system, Big data, Wheat genomics, genetics and phenomics

## Abstract

The Wheat@URGI portal has been developed to provide the international community of researchers and breeders with access to the bread wheat reference genome sequence produced by the International Wheat Genome Sequencing Consortium. Genome browsers, BLAST, and InterMine tools have been established for in-depth exploration of the genome sequence together with additional linked datasets including physical maps, sequence variations, gene expression, and genetic and phenomic data from other international collaborative projects already stored in the GnpIS information system. The portal provides enhanced search and browser features that will facilitate the deployment of the latest genomics resources in wheat improvement.

**Electronic supplementary material:**

The online version of this article (10.1186/s13059-018-1491-4) contains supplementary material, which is available to authorized users.

## Background

The International Wheat Genome Sequencing Consortium (IWGSC) [1] is an international collaborative group of growers, academic scientists, and public and private breeders that was established to generate a high-quality reference genome sequence of the hexaploid bread wheat, and to provide breeders with state-of-the-art tools for wheat improvement. The vision of the consortium is that the high-quality, annotated ordered genome sequence integrated with physical maps will serve as a foundation for the accelerated development of improved varieties and will empower all aspects of basic and applied wheat science to address the important challenge of food security. A first analysis of the reference sequence produced by the consortium (IWGSC RefSeq v1.0) was recently published [2].

To ensure that wheat breeding and research programs can make the most of this extensive genomic resource, the IWGSC endorsed the establishment of a data repository at URGI (Unité de Recherche Génomique Info/research unit in genomics and bioinformatics) from INRA (Institut National de la Recherche Agronomique/French national institute for agricultural research) to develop databases and browsers with relevant links to public data available worldwide. The IWGSC data repository is thus hosted by URGI to support public and private parties in data management as well as analysis and usage of the sequence data. Wheat functional genomics (expression, methylation, etc.), genetic, and phenomic data have increased concurrently, requiring the development of additional tools and resources to integrate different data for biologists and breeders. To manage this escalation of data, URGI has built this data repository for the wheat community with the following specific aims: (1) to store resources for which no public archive exists (e.g. physical maps, phenotype information); (2) to enable pre-publication access to specific datasets (e.g. sequence assemblies and annotations, physical maps, markers); and (3) to enable rapid release of integrated resources upon publication. The repository has been designed in accordance with the “FAIR” principles [3] to ensure that the data are Findable, Accessible, Interoperable, and Reusable. To address the challenge of integrating diverse data types from multiple sources, URGI employs solutions that provide enhanced features for data exploration, mining, and visualisation using the GnpIS information system [4] combined with a high level of data interoperability.

Here we describe the data and tools currently available through the Wheat@URGI portal [5], the primary resource for the reference sequence of the bread wheat genome (IWGSC RefSeq v1.0) and other IWGSC wheat genomic data. The links to functional genomics, genetic, and phenomic data from many other large wheat projects are also described.

## A wealth of data is available throughout the Wheat@URGI portal

The data hosted by the Wheat@URGI portal are available through flat files stored in the IWGSC data repository and through the GnpIS information system [4]. GnpIS encompasses a set of integrated databases to manage genomic data using well-known tools such as the Basic Local Alignment Search Tool (BLAST), JBrowse, GBrowse, and InterMine, and an in-house database called GnpIS-coreDB developed by URGI to manage genetic and phenomic data.

### IWGSC data

Through its concerted efforts to achieve a high-quality, functionally annotated reference wheat genome sequence, the IWGSC has developed a variety of resources for the bread wheat (*Triticum aestivum* L.) accession Chinese Spring. The IWGSC data hosted in the Wheat@URGI portal within the IWGSC data repository are shown in Table 1. They fall into four broad categories: (1) physical maps, (2) sequence assemblies and annotations, (3) gene expression data, and (4) variation data.

**Table 1:** IWGSC open access data summary hosted in the IWGSC data repository of the Wheat@URGI portal in July 2018

Data	Details	Tools	Contacts
IWGSC RefSeq v1.0 assembly	Scaffolds, super scaffolds, pseudomolecules	Download, BLAST, browser, and InterMine	IWGSC
IWGSC RefSeq v1.0 annotation	Genes, transposable elements, non-coding RNAs, markers, functional annotation, varietal SNPs, GBS maps, optical maps, Radiation Hybrid maps	Download, browser, and InterMine	IWGSC
IWGSC RefSeq v1.1 annotation	Genes, RNA-Seq mapping	Download, browser, and InterMine	IWGSC
IWGSC WGA v0.4 assembly	Scaffolds, superscaffolds, pseudomolecules	Download and BLAST	IWGSC
IWGSC Survey sequence v2 assembly and annotation	Contigs, gene models, markers, Genome Zipper, POPSEQ	Download, BLAST, and browser	IWGSC, Mihaela Martis, Manuel Spannagl, Klaus Mayer, Nils Stein, Curtis Pozniak, Eduard Akhunov
IWGSC Survey sequence v3 assembly and annotation	Scaffolds, gene models	Download, BLAST, and browser	Andy Sharpe, David Konkin, Curtis Pozniak
3B reference sequence assembly and annotation	Contig, scaffolds, pseudomolecule, genes, transposable elements, RNAs, markers	Download, BLAST, and browser	Frédéric Choulet, Etienne Paux
Other wheat species WGS assemblies	*Triticum durum* cv. Cappelli, *Triticum durum* cv. Strongfield, *Triticum monococcum*, *Aegilops speltoides*, *Aegilops sharonensis*, *Triticum urartu*, *Aegilops tauschii*	Download and BLAST	Jon Wright, Mario Caccamo
Expression	Deep transcriptome sequencing	Download	Lise Pingault, Etienne Paux
	*Triticum urartu* and *Triticum turgidum* (GrainGenes)	Download	Jorge Dubcovsky
Variations	Varietal SNPs	Download and browser	Jorge Dubcovsky, Eduard Akhunov
	IWGSC SNPs	Download	Etienne Paux
	GBS and Whole Exome Capture	Download	Eduard Akhunov
Physical maps	1AS v1 and v2	Download and browser	James Breen, Thomas Wicker, Beat Keller
	1AL v1 and v2	Download and browser	Stuart Lucas, Hikmet Budak
	2AS	Download and browser	Kuldeep Singh
	2AL	Download and browser	Kuldeep Singh
	3AS v1	Download and browser	Sunish Sehgal, Bikram Gill
	3AS v2	Download and browser	Sunish Sehgal, Bikram Gill
	3AL	Download and browser	Vijay Kumar Tiwari
	4AS	Download and browser	Miroslav Valarik, Jaroslav Dolezel
	4AL v1 and v2	Download and browser	Miroslav Valarik, Jaroslav Dolezel
	5AS	Download and browser	Simone Scalabrin
	5AL	Download and browser	Simone Scalabrin
	6AS	Download and browser	Naser Poursarebani
	6AL	Download and browser	Naser Poursarebani
	7AS	Download and browser	Gabriel Keeble-Gagnere
	7AL	Download and browser	Gabriel Keeble-Gagnere
	1BS v1, v2, v3, and v5	Download and browser	Dina Raats, Zeev Frenkel, Abraham Korol
	1BL v1 and v2	Download and browser	Etienne Paux
	2BS	Download and browser	John Jacobs
	2BL	Download and browser	John Jacobs
	3B	Download and browser	Etienne Paux
	4BS	Download and browser	John Jacobs
	4BL	Download and browser	John Jacobs
	5BS	Download and browser	Elena Salina
	5BL	Download and browser	John Jacobs
	6BS v1 and v2	Download and browser	Fuminori Kobayashi, Hirokazu Handa
	6BL v1 and v2	Download and browser	Fuminori Kobayashi, Hirokazu Handa
	7BS	Download and browser	Tatiana Belova, Odd-Arne Olsen
	7BL	Download and browser	Tatiana Belova, Odd-Arne Olsen
	1D	Download and browser	Bikram Gill, Sunish Sehgal, Vijay Kumar Tiwari
	2DS	Download and browser	John Jacobs
	2DL	Download and browser	John Jacobs
	3DS v1 and v2	Download and browser	Jan Bartos, Jaroslav Dolezel
	3DL	Download and browser	Jon Wright, Mario Caccamo, Mike Bevan
	4D	Download and browser	Bikram Gill, Sunish Sehgal, Vijay Kumar Tiwari
	5DS	Download and browser	Hikmet Budak, Bala Ani Akpinar
	5DL	Download and browser	John Jacobs
	6D	Download and browser	Bikram Gill, Sunish Sehgal, Vijay Kumar Tiwari
	7DS	Download and browser	Hana Simkova, Jaroslav Dolezel
	7DL	Download and browser	Song Weining, Wang Le

Enquiries about these data should be addressed to communications@wheatgenome.org and urgi-contact@inra.fr

#### Physical maps

Physical maps assembled by IWGSC scientists for the 21 bread wheat chromosomes, based on high-information-content (fluorescence) fingerprinting (HICF) [6] or Whole Genome Profiling (WGP™) [7] of flow-sorted chromosome or chromosome-arm specific bacterial artificial chromosome (BAC) libraries, are stored and displayed. The positions of individual BAC clones, markers, and deletion bins are mapped onto physical contigs. The database maintains all released versions of each physical map with the software used to produce the BAC clone assemblies (FingerPrinted Contig (FPC) [8] or Linear Topological Contig (LTC) [9]), information from the group that produced the map, and a link to order the BAC clones from the French plant genomic resource centre [10].

#### Sequence assemblies and annotations

The IWGSC wheat genome sequence assemblies available for download, BLAST [11], and display in genome browsers include the draft survey sequence assemblies released in 2014 (IWGSC Chromosome Survey Sequencing (CSS) v1) and two improved versions (CSS v2 and v3) [12] and the chromosome 3B reference sequence (the first reference-quality chromosome sequence obtained by the consortium) [13]. Associated with these assemblies are the virtual gene order map generated for the CSS (Genome Zipper), the population sequencing (POPSEQ) data used to order sequence contigs on chromosomes [14], and mapped marker sets. The reference sequence of the bread wheat genome (IWGSC RefSeq v1.0, 14.5 Gb assembly with super scaffold N50 of 22.8 Mb) was obtained by integrating whole genome shotgun Illumina short reads assembled with NRGene’s DeNovoMAGIC™ software with the wealth of IWGSC map and sequence resources [2]. IWGSC RefSeq v1.0 is available for download, BLAST, and browser display. Users can access the whole genome, pseudomolecules of individual chromosomes or chromosome arms, and scaffolds with the structural and functional annotation of genes, transposable elements, and non-coding RNAs generated by the IWGSC. In addition, mapped markers as well as alignments of nucleic acid and protein evidence supporting the annotation are available. Updated versions of the annotation for genes belonging to specific gene families or regions of specific chromosomes that have been manually annotated (ca. 3685 genes) can be found in the IWGSC RefSeq v1.1 annotation.

In addition to the bread wheat sequence, the IWGSC also assembled seven diploid and tetraploid wheat-related species: *Triticum durum* cv. Cappelli, *Triticum durum* cv. Strongfield, *Triticum durum* cv. Svevo, *Triticum monococcum*, *Triticum urartu*, *Aegilops speltoides*, *Aegilops sharonensis* [12]. Download and BLAST are available for these data.

#### Expression data

RNA-Seq expression data are available as read counts and transcripts per kilobase million (TPM) for the IWGSC RefSeq v1.1 annotation. It is a transcriptome atlas developed from 850 RNA-Seq datasets representing a diverse range of tissues, development stages, and environmental conditions [15].

#### Variation data

These data consist of downloadable variant call format (VCF) files from genotyping by sequencing (GBS) and whole exome capture experiments of 62 diverse wheat lines [16] and of the IWGSC 3,289,847 single nucleotide polymorphisms (SNPs) [17]. Moreover, varietal SNPs aligned on IWGSC RefSeq v1.0 can be displayed in the browser and downloaded.

### Wheat gene pool

As well as IWGSC resources, URGI also hosts other open access wheat sequence data to facilitate research into the wheat gene pool. Sequence assemblies available for download and BLAST include the bread wheat whole genome sequence assembly *Triticum aestivum* TGACv1 [18] and the diploid progenitor of *Aegilops tauschii* [19].

### Genetic and phenomic data

In addition to sequence data, the Wheat@URGI portal hosts, within GnpIS-coreDB, several sets of genetic and phenomic wheat data [20] that have been produced from French, European, and international projects since 2000 [21]. A significant amount of these data is available without restriction. However, access to restricted data can be obtained through a material transfer or intellectual property agreement. Table 2 presents the types and number of genetic and phenomic data hosted in the GnpIS-coreDB database.

**Table 2:** Genetic and phenomic wheat data summary hosted in the GnpIS-coreDB database of the Wheat@URGI portal in March 2018

Data type	Object	Total number	No. open access	No. restricted access to projects
Genetic resources	Taxon	56	56	0
	Accession	12,839	10,016	2823
Genetic maps	Map	30	29	1
	Marker	704,822	34,164	670,658
	QTL	749	465	284
SNP discovery	Sequence Variation	4,189,312,581	90	4,189,312,491
	SNP, indel	724,132	95	724,037
Genotyping (high throughput)	Experiment	23	2	21
	Sample	8885	47	8872
	Marker	668,540	0	668,540
Phenotyping	Trial	850	821	29
	Plot	3660	2985	901
	Variable	282	89	195
	Observation	1,171,172	527,981	643,191
GWAS	Analysis	1555	43	1512
	Sample	2365	1839	526
	Variable	359	37	322
	Marker	123,866	4109	119,757
	Association	824,217	48,596	775,621

Questions about these data can be addressed to urgi-contact@inra.fr

Genetic information corresponds to genetically mapped markers, quantitative trait loci (QTLs), genetic resources (germplasms), and genetic studies (genome-wide association studies (GWASs)). Genomic information consists of variations from SNP discovery experiments, genotyping, comparative genomics (synteny), and expression data (microarray, RNA-Seq). Phenomic data are available as whole trials including phenotypic and environmental observations recorded using variables from ontologies with Minimum Information About a Plant Phenotyping Experiment (MIAPPE) [22] compliant metadata.

Germplasm data were mainly provided by the French small grain cereals genebank maintained by INRA at Clermont-Ferrand [23] but also by partners of several European Union (EU) projects. They were linked together with related genotyping or phenotyping characterisation data. Generally, genetic and phenomic data have been produced by INRA and its partners in large collaborative projects.

## Browsing and searching a large variety of integrated data

Data can be easily accessed through the Wheat@URGI portal [5] using (1) tabs at the top of the webpages allowing access in one click to the data, tools, and projects descriptions as well as the IWGSC data repository, (2) direct links from the home page to the different data types (e.g. clicking on “Physical maps” opens the physical maps browser), and (3) data discovery and InterMine [24] tools on the home page.

The IWGSC data repository [25] allows one to access consortium data by (1) clicking on a chromosome to open a pop-up menu with all related data (e.g. 3A, 3B, etc.), or (2) using the tabs on the left to access the data by type (e.g. Assemblies, Annotations, etc.) or useful links to the news, the BLAST tool, frequently asked questions, the access status of the data (e.g. open access), etc.

### Physical maps browser

GBrowse [26] displays the physical maps generated by the IWGSC members [27]. A clickable image on the top of the browser gives access to all versions of the physical map for each chromosome. The browser displays physical contigs, BACs, deletion bins, and markers. From the BACs track, it is possible to order BAC clones directly at the INRA French plant genomic resource centre [10]. From the BACs and markers tracks, one can go directly to the corresponding region in the IWGSC RefSeq v1.0 browser.

### Genome browser and BLAST

IWGSC RefSeq v1.0 is displayed in a dedicated JBrowse [28, 29]. The “markers track” provides links to additional genetic information stored in GnpIS-coreDB which includes access to the position of the marker in centimorgans (cM) on genetic maps and to the overlapping QTLs. The most popular tool of the IWGSC data repository is the BLAST search tool (476,000 BLAST searches launched in 2017, Additional file 1: Table S1, Additional file 1: Figure S1). All of the wheat sequences available on the Wheat@URGI portal are indexed for BLAST search (see [30] for the complete list). A set of databanks can be selected, e.g. IWGSC RefSeq v1.0 and IWGSC CSS v3 for a given chromosome. The result is presented in a classical tabular format with (1) links to download the data (matching contigs and high scoring pairs (HSP)), (2) links on the genome browsers directly zooming in on the matching region, and (3) external links to Ensembl Plants [31].

### Genetic and phenomic data in GnpIS-coreDB

The IWGSC sequence data are linked to genetic and phenomic data within the GnpIS information system [4]. This integration is organised around key data, also called “pivot data” as they are pivotal objects which allow integration between data types. The key objects used to link genomic resources to genetic data are markers and QTLs. Markers are mapped on the genome sequences and provide information on neighbour genes and their function. They also have links to GnpIS-coreDB genetic maps, QTLs, genotyping, and GWAS data. Additional information on the marker itself can be found regarding the marker type (e.g. simple sequence repeats (SSRs), Diversity Arrays Technology (DArT)), the primer sequences for PCR amplification, and SNP details (including the flanking sequences) when relevant. QTLs link the genetic data to the phenomic data in GnpIS-coreDB and to synteny data displayed by the PlantSyntenyViewer tool [32, 33].

The accessions (i.e. germplasm) and the variables (i.e. observed traits), described with dedicated ontologies, are other important key data for genetic studies as they allow linking phenotype data to genetic associations and QTLs through traits. The genetic resources stored in GnpIS-coreDB displays the unambiguous identification of the accession used (with Digital Object Identifier (DOI)) and a rich set of associated data following the Multi-Crop Passport Descriptors (MCPD, [34]) standard: picture, synonyms, descriptors, geolocation of the sites (origin, collecting, and evaluation), the collections or panels it belongs to, and the holding stock centre with a link to order the accession when possible. The phenotype data include traceability on trials with timing (e.g. year, temporal series), location, and environment including soil and cultural practices. The phenotype and environment variables follow the Crop Ontology format [35], which includes unique identifiers for each variable, composed of a trait (e.g. grain yield, plant height, spike per plant, etc.), a method (e.g. measurement, computational), and a scale (e.g. International System of Units, notation scale). All these data are displayed in the GnpIS-coreDB web interface and can be downloaded in different file formats, all compliant with the MIAPPE standard [22].

### Mining and data discovery tools

To complete these already rich integrated datasets, a gene-centric data warehouse, the WheatMine, has been set up using the well-established InterMine tool [24]. The gene card displays gene function, gene ontology terms, and overlapping genomic features. WheatMine [36] provides access to the IWGSC RefSeq v1.0 and v1.1 annotation data (genes, messenger RNA, polypeptides, transposable elements), markers and, through pivotal objects, to genetic data (QTL, metaQTL). It is also possible to navigate from a gene card to its position on the wheat genome browser or to relevant marker details in GnpIS-coreDB.

**Fig. 1:**
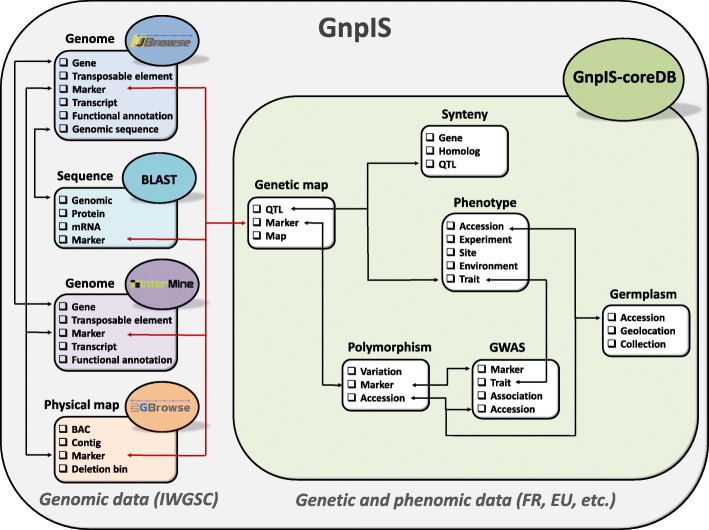
Conceptual view of wheat data links in GnpIS. *Arrows* illustrate existing links between data types allowing data integration. *Red arrows* highlight links between genomics and genetics

Figure 1 summarises the concept and the tools to navigate through the key data in GnpIS.

Finally, to facilitate data search and access to this wealth of data, we developed a data discovery tool, which, similar to a google search, allows the user to enter keywords or terms to find all the matching information in the various data warehouses. The results are presented in a table with details on the matches (database source, type, species, description) and a direct link to the feature (e.g. a gene in a browser, a marker page in GnpIS-coreDB, etc.).

**Fig. 2:**
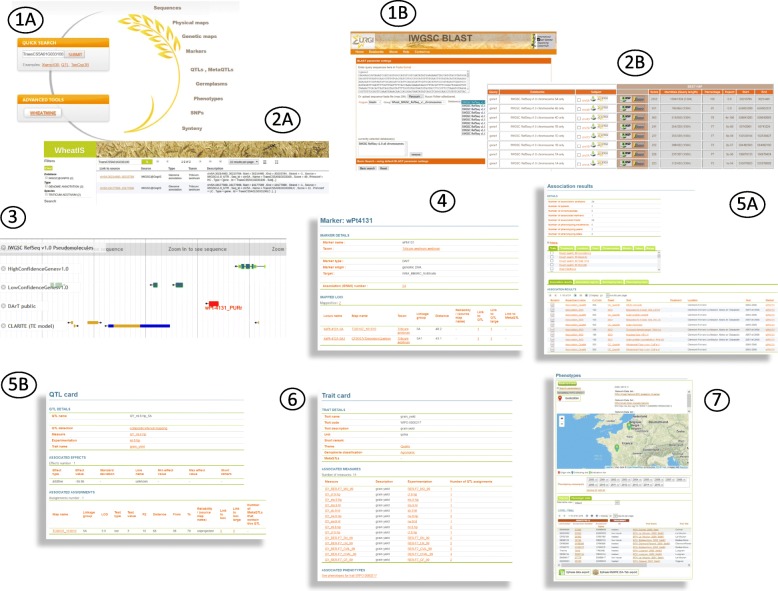
Screenshots of the web interfaces for a practical use case to explore all the genomic data in the vicinity of a dedicated gene and find out if there are genetic studies pointing to this genomic region. Search the gene name (e.g. *TraesCS5A01G033100*) in the data discovery tool (1A, [5]) or BLAST the sequence of the gene against IWGSC RefSeq v1.0 (1B, [30]). The results are displayed in Tables (2A, 2B) with links to JBrowse directly zooming in on the gene [48]. Explore the region around the gene to find a marker (3, e.g. wPt-4131_PURr). By clicking on the marker, display additional information stored in GnpIS-coreDB (4, [49]) showing that the marker is used in GWAS experiments (5A, [50]) and is linked to a QTL (5B, e.g. GY_ml.8.Np_5A, [51]). From the trait description of this QTL (6, [52]), display all the phenotyping experiments performed on this trait (7, e.g. grain yield, [53])

Figure 2 highlights a practical use case describing how to use the Wheat@URGI portal to go from a gene sequence to find the related genetic studies.

## Conclusion and future directions

The Wheat@URGI portal hosts and gives access to essential, high-quality wheat data from the IWGSC, European, and international projects. Furthermore, its added value is that it integrates different data types altogether (genomics, genetics, and phenomics) and provides dedicated tools to explore them.

As new wheat resources such as GWASs, genomic selection, and pan-genome data are generated in the frame of ongoing projects, GnpIS will allow their management and integration with other data already available in the information system, linking new upcoming data to this central IWGSC genomic resource.

At a wider scale, an expert working group (EWG) of the international Wheat Initiative has built an international wheat information system, called WheatIS, with the aim of providing a single-access web-based system to all available wheat data resources and bioinformatics tools [37]. The Wheat@URGI portal is a major node of the WheatIS federation that exposes genomic, genetic, and phenomic integrated data to the community. The WheatIS data discovery tool allows a one-stop search in GnpIS [4] (including IWGSC browsers, InterMine and GnpIS-coreDB), from URGI; Ensembl Plants, from the European Bioinformatics institute (EMBL-EBI) [31]; CrowsNest [38], at the Plant Genome and Systems Biology (PGSB) group; CR-EST [39], GBIS [40] and MetaCrop [41], from the Leibniz Institute of Plant Genetics and Crop Plant Research (IPK); The Triticeae Toolbox (Triticeae Coordinated Agricultural Product); CIMMYT DSpace and Dataverse (International Maize and Wheat Improvement Center (CIMMYT)); Gramene [42], from Cold Spring Harbor Laboratory (CSH), Ohio State University (OSU), and EMBL-EBI; Cropnet, from the Institute of Plant Genetics of the Polish Academy of Sciences (IPGPAS); WheatPan [43], from the University of Western Australia (UWA); and GrainGenes [44], US Department of Agriculture (USDA).

**Fig. 3:**
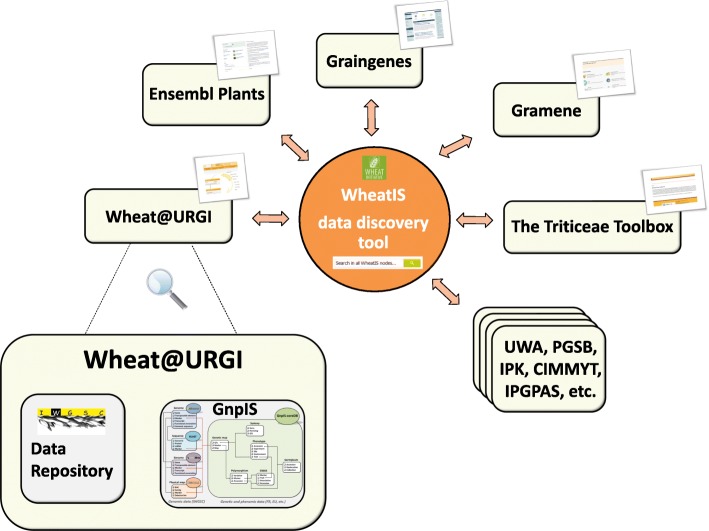
The Wheat@URGI portal node in the WheatIS ecosystem. *Boxes* represent the different information systems queried by the WheatIS data discovery tool

Figure 3 presents the WheatIS ecosystem.

Data integration is fundamental for researchers and breeders who want to use genomic information to improve wheat varieties. However, the diversity of data types and the concomitant lack of data harmonisation and standards hamper cross-referencing and meta-analysis. A joint action between the WheatIS EWG and a group of linked data scientists created the Wheat Data Interoperability Working Group under the Research Data Alliance (RDA) umbrella [45] to help tackle this difficult issue [46]. The Wheat@URGI portal continuously evolves its repository to follow the standard recommendations [47].

## Additional file

Additional file 1: Supplementary data on software technologies and usage metrics. (PDF 541 kb)

## Abbreviations

BAC: Bacterial artificial chromosome
BLAST: Basic Local Alignment Search Tool
CIMMYT: International Maize and Wheat Improvement Center
cM: Centimorgan
CSH: Cold Spring Harbor Laboratory
CSS: Chromosome Survey Sequencing
DArT: Diversity Arrays Technology
DOI: Digital object identifier
EMBL-EBI: European Bioinformatics Institute
EWG: Expert working group
FAIR: Findable, accessible, interoperable, reusable
FPC: FingerPrinted Contig
GBS: Genotyping by sequencing
GWAS: Genome-wide association study
HICF: High-information-content fingerprinting
HSP: High scoring pairs
INRA: Institut National de la Recherche Agronomique/French national institute for agricultural research
IPGPAS: Institute of Plant Genetics of the Polish Academy of Sciences
IPK: Leibniz Institute of Plant Genetics and Crop Plant Research
IWGSC: International Wheat Genome Sequencing Consortium
LTC: Linear topological contig
MCPD: Multi-Crop Passport Descriptor
MIAPPE: Minimum Information About a Plant Phenotyping Experiment
OSU: Ohio State University
PCR: Polymerase chain reaction
PGSB: Plant Genome and Systems Biology (group)
POPSEQ: Population sequencing
QTL: Quantitative trait locus
RDA: Research Data Alliance
RNA: Ribonucleic acid
SNP: Single nucleotide polymorphism
SSR: Simple sequence repeat
TPM: Transcripts per kilobase million
URGI: Unité de Recherche Génomique Info/research unit in genomics and bioinformatics
USDA: US Department of Agriculture
UWA: University of Western Australia
VCF: Variant call format
WGP™: Whole genome profiling
