# Supplementary material for: Linking the International Wheat Genome Sequencing Consortium bread wheat reference genome sequence to wheat genetic and phenomic data
Source: Genome Biol. 2018 Aug 17;19:111. doi: 10.1186/s13059-018-1491-4 (PMC6097284; doi:10.1186/s13059-018-1491-4)
Supplement: Supplementary file 1 — Supplementary data on software technologies and usage metrics. (PDF 541 kb) [file 13059_2018_1491_MOESM1_ESM.pdf]

Supplementary data

Software technologies

The Wheat@URGI portal website is based on eZ Publish v4 open source content management system (https://ez.no/) using the PHP language and a MySQL database (https://www.mysql.com/).

The genome browsers are based on the GMOD (http://gmod.org/wiki/Main_Page) GBrowse v2.33 [26] and JBrowse v1.11.5 [28] built with JavaScript and HTML5. We customized GBrowse to display the physical map data. The gff3 file is generated from the .fpc file obtained by the data producer using the FPC [8] or the LTC [9] tools.

The stand-alone BLAST web interface implemented at URGI is based on ViroBLAST [48], customized to obtain a user-friendly grouping of searched databanks and visualization of the results. A robust file download system was also developed using a home-made php script to handle big data volume.

GnpIS-coreDB is a URGI development using state of the art technologies: Java EE framework (http://www.oracle.com/technetwork/java/javaee/overview/index.html), GWT (Google Web Toolkit, http://www.gwtproject.org/), Spring boot v1.4 (https://projects.spring.io/spring-boot/), PostgreSQL relational database v9.6 (https://www.postgresql.org/) and Elasticsearch NoSQL database v2.3.3 (https://www.elastic.co/). To set-up a GnpIS-coreDB dedicated to the wheat community, a filter allowing to display only the wheat data (*Triticum*, *Aegilops*) and barley data (*Hordeum*) was developed. This filter relies on a variable-length multidimensional arrays field in the PostgreSQL database. It is completely transparent to the user and allows him to navigate in GnpIS-coreDB through wheat data only. New versions of the GnpIS-coreDB software are deposited in the APP, the European body for protecting authors and publishers of digital works (http://www.app.asso.fr/en/welcome.html).

WheatMine uses InterMine [24] v1.8.3 which provides a fast, flexible and user friendly access to integrated data by multiple ways: a browser, a query builder and a region search tool. Users can filter their favorite features, save their own queries, and export results in many different formats (GFF3, BED or XML). An On-line documentation and pre-computed queries are also available.

The data discovery tool relies on the Solr full-text indexing technology v6.6.2 (http://lucene.apache.org/solr/). We used a restriction on the wheat and barley species to search only the corresponding data in the indexed databases. The tool was packaged and is downloadable (https://wheat-urgi.versailles.inra.fr/Projects/Wheat-Information-System/SolR-tool-package).

Usage metrics

Table S1. Usage statistics of genomics data in the Wheat@URGI portal (all numbers exclude web-robots and internal IP).

Number of	2012	2013	2014	2015	2016	2017
Visits on the IWGSC Sequence Repository website	N/A	11440	20754	27070	20841	28151
Downloads of wheat sequence data	2253	4413	17783	19307	18724	22935
Visits on the wheat browsers	5869	9370	9130	22989	22373	18262
Number of BLAST searches on wheat sequences	14100	60400	86000	92446	195763	476595
Number of WheatIS data discovery tool searches	N/A	N/A	N/A	N/A	13010	26480

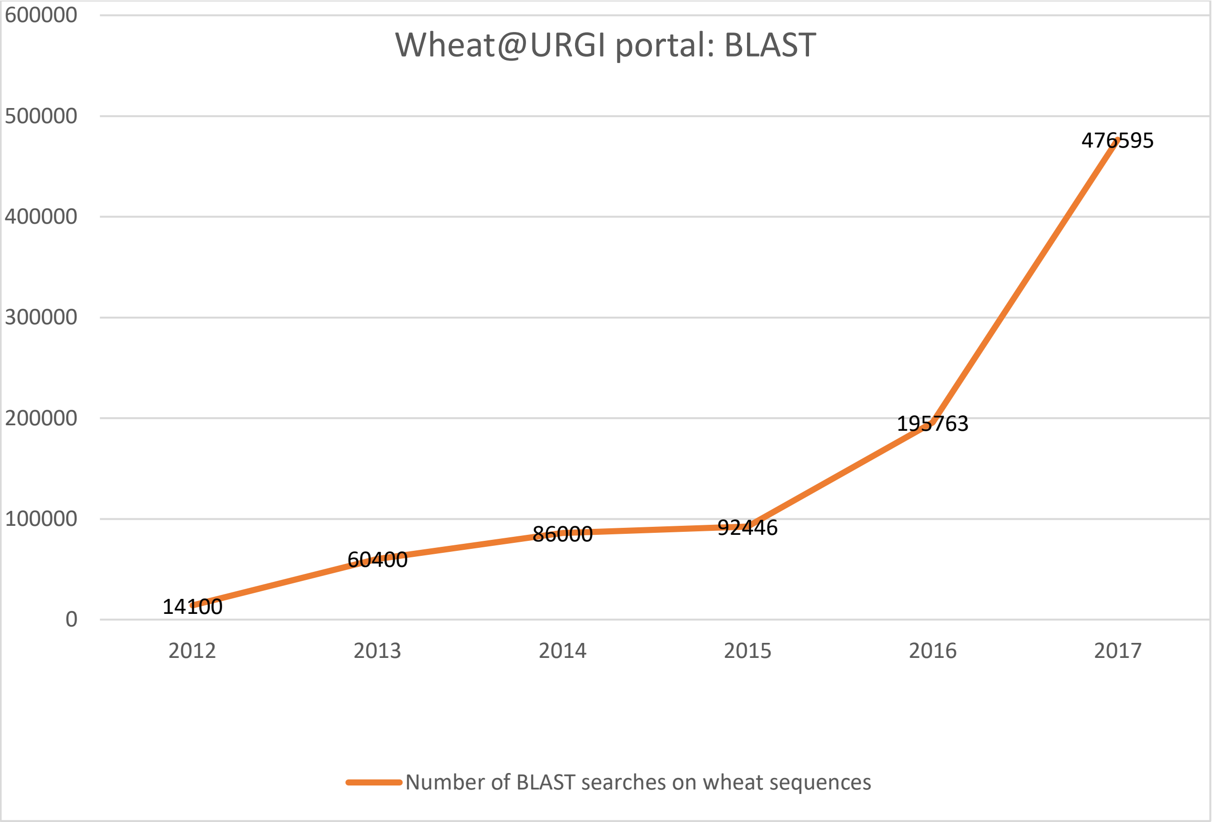

Figure S1. Focus on the BLAST usage.
